# Loss of c-Met Disrupts Gene Expression Program Required for G2/M Progression during Liver Regeneration in Mice

**DOI:** 10.1371/journal.pone.0012739

**Published:** 2010-09-16

**Authors:** Valentina M. Factor, Daekwan Seo, Tsuyoshi Ishikawa, Pal Kaposi-Novak, Jens U. Marquardt, Jesper B. Andersen, Elizabeth A. Conner, Snorri S. Thorgeirsson

**Affiliations:** Laboratory of Experimental Carcinogenesis, Center for Cancer Research, National Cancer Institute, National Institutes of Health, Bethesda, Maryland, United States of America; The University of Hong Kong, Hong Kong

## Abstract

**Background:**

Previous work has established that HGF/c-Met signaling plays a pivotal role in regulating the onset of S phase following partial hepatectomy (PH). In this study, we used *Met^fl/fl^;Alb-Cre^+/−^* conditional knockout mice to determine the effects of c-Met dysfunction in hepatocytes on kinetics of liver regeneration.

**Methodology/Principal Finding:**

The priming events appeared to be intact in *Met^fl/fl^;Alb-Cre^+/−^* livers. Up-regulation of stress response (*MAFK*, *IKBZ*, *SOCS3*) and early growth response (*c-Myc*, *c-Jun*, *c-Fos*, *DUSP1* and *6*) genes as assessed by RT-qPCR and/or microarray profiling was unchanged. This was consistent with an early induction of MAPK/Erk and STAT3. However, after a successful completion of the first round of DNA replication, c-Met deficient hepatocytes were blocked in early/mid G2 phase as shown by staining with phosphorylated form of histone H3. Furthermore, loss of c-Met in hepatocytes diminished the subsequent G1/S progression and delayed liver recovery after partial hepatectomy. Upstream signaling pathways involved in the blockage of G2/M transition included lack of persistent Erk1/2 activation and inability to up-regulate the levels of Cdk1, Plk1, Aurora A and B, and Mad2 along with a defective histone 3 phosphorylation and lack of chromatin condensation. Continuous supplementation with EGF *in vitro* increased proliferation of *Met^fl/fl^;Alb-Cre^+/−^* primary hepatocytes and partially restored expression levels of mitotic cell cycle regulators albeit to a lesser degree as compared to control cultures.

**Conclusion/Significance:**

In conclusion, our results assign a novel non-redundant function for HGF/c-Met signaling in regulation of G2/M gene expression program via maintaining a persistent Erk1/2 activation throughout liver regeneration.

## Introduction

Liver regeneration after partial hepatectomy (PH) is a process of compensatory hyperplasia which involves all cell types within the remaining liver lobes and depends on the multitude of interrelated regulatory pathways that directly or indirectly control successful restoration of hepatic mass [1]–[3]. The major conceptual advances as set forth by Nelson Fausto categorize the signaling pathways into cytokine, growth factor and metabolic networks integrating reparative proliferation with continuation of hepatic function [2]. According to the model, liver regeneration starts from the activation of immediate early response genes driven primarily by tumor necrosis factor-α (TNF-α) and interleukin 6 (IL-6), which are produced by nonparenchymal cells and prime quiescent hepatocytes for the transition into G_1_ stage. Among the major growth factors involved at this stage is the hepatocyte growth factor (HGF) [4].

HGF is a mesenchyme-derived multifunctional molecule that elicits its activities through a single receptor c-Met [5], [6]. Due to the multitude of HGF/c-Met functions, including regulation of mitogenesis, motogenesis, and morphogenesis, HGF signaling is considered to be a primary stimulator of liver regeneration. Accordingly, hepatic overexpression of HGF remarkably accelerated whereas loss of c-Met signaling decreased regenerative potential of liver [7]–[10].

A key step in c-Met signaling is HGF-induced phosphorylation of the kinase domain followed by autophosphorylation and activation of numerous downstream effectors [11], [12]. The best known for its role in the initiation of S phase is mitogen-activated protein kinase (MAPK)/Erk pathway. Gene knockout studies in rodents confirmed the central role of c-Met in cell cycle initiation and progression. Recent work by Borowiak and colleagues has shown that conditional inactivation of *c-met* using a ubiquitously expressed alpha/beta interferon-inducible promoter resulted in suppressed Erk1/2 activation and defective exit from quiescence in regenerating mouse liver [7]. Similarly, Michalopoulos' group found a blocked entry into the cell cycle and increased apoptosis during the first 24 hr of rat liver regeneration by silencing *c-met* via RNA interference [9]. Both approaches caused inactivation of *c-met* in diverse liver cell types, and therefore the extent to which the lack of c-Met signaling specifically in hepatocytes contributed to impaired cell cycle regulation could not be elucidated. Further, although prior evidence implicated Erk1/2 pathway in regulating G1/S transition, its role during G2/M transition and how it relates to c-Met function remains to be fully realized.

To address these questions, we designed our experiments using two c-Met conditional knockout models: a hepatocyte- and liver specific mutant mice in which the exon encoding the ATP-binding site of *c-met* was deleted either by a constitutive *Alb-Cre* or inducible *Mx1-Cre* transgenes. Here we report for the first time that besides its role in G1/S progression, c-Met is essential regulator of G2/M progression through a mechanism involving Erk1/2-dependent activation of key mitotic regulators including Cdk1, Plk1, and Aurora A and B.

## Results

### c-Met is not Required for Postnatal Liver Growth

To elucidate the mechanism of growth regulation by c-Met specifically in hepatocytes, we used a hepatocyte-specific c-Met conditional knockout mouse model described previously [8]. In these mice, the deletion of the exon encoding the ATP-binding site of *c-met* is achieved by constitutively active *Alb-Cre* transgene and thus restricted to hepatocytes whereas nonparenchymal liver cells are not targeted. The lack of c-Met expression in hepatocytes was confirmed by PCR analysis and immunohistochemistry (Figure S1A and B). Mice with a selective disruption of *c-met* in hepatocytes appeared normal without apparent histological abnormalities. Although liver mass was slightly reduced, there were no differences in blood parameters or ploidy as estimated at 2 months of age, indicating that c-Met deficiency did not affect the postnatal liver growth (Figure S1D and E).

### Loss of c-Met Delays Recovery of Liver Mass after Partial Hepatectomy

To investigate the requirement for c-Met function during liver regeneration, we performed a standard 70% hepatectomy using *Met^wt/wt^;Alb-Cre^+/−^* and *Met^fl/fl^;Alb-Cre^+/−^* mice. Even though *Met^fl/fl^;Alb-Cre^+/−^* mice were more sensitive to metafane anesthesia [8], there was no difference in the accumulated postoperative survival during the first week after surgery when we used a mixture of isoflurane/oxygen for anesthesia and kept the gall bladder intact (96.9% in control versus 94.3% and c-Met mutant mice). The serum levels of aspartate aminotransferase were also not different at 24 hr and 48 hr after PH reflecting a comparable tissue injury in mice of both genotypes (Figure S2A). Histological examination of the regenerative livers also revealed neither increased cell death nor presence of necrosis suggesting that loss of c-Met function did not interfere with hepatocyte survival after PH. Nonetheless, a regeneration index calculated as an increase in liver-to-body mass ratio was lower in c-Met mutant mice and reached only 77.2±4.3% of the original liver mass after 168 hr as compared to 93.6±3.3% in controls (Figure S2B). Blood levels of albumin and triglyceride were also decreased indicating that c-Met knockout mice could not regenerate liver mass and function efficiently (Figure S2C and D).

### c-Met Regulates both G1/S and G2/M Transitions

**Figure 1:**
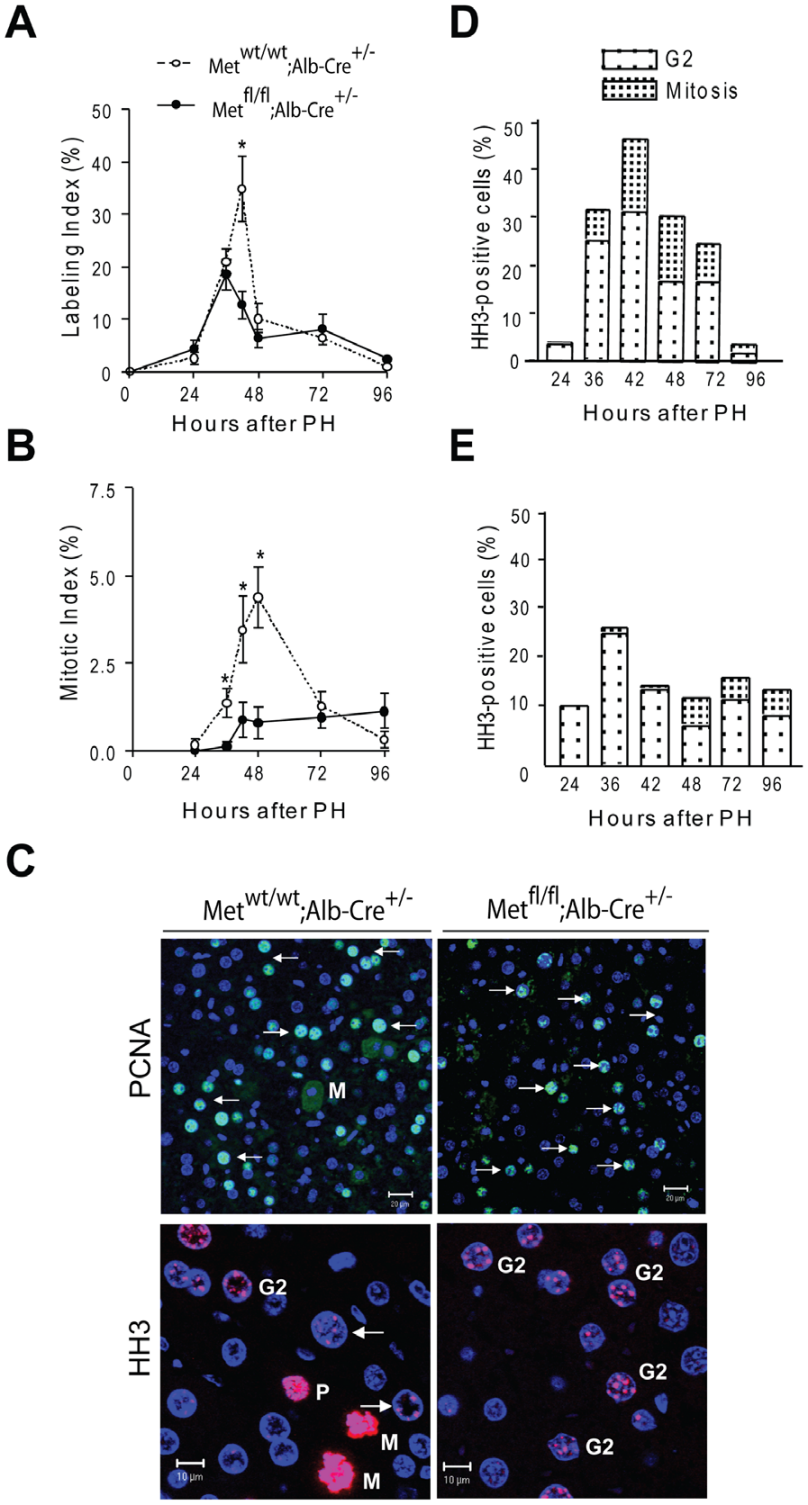
Cell cycle progression defects in c-Met-deficient regenerating livers. (**A**) Normal induction of the first but reduced second wave of DNA replication and (**B**) inhibition of mitosis in *Met^fl/fl^;Alb-Cre^+/−^* livers. The labeling and mitotic indexes were calculated by randomly counting BrdU-stained nuclei (**A**) and mitotic figures (**B**) in a total of 2,000 hepatocytes and expressed as percent. Results shown are the means ± SE (n = 5−7 mice/group/time point). Asterisks indicate statistical significance assessed by Student's *t*- test (*P<0.05*). (**C**) Representative photomicrographs of PCNA (top) and phosphor-histone H3 staining of liver sections. Shown are cells in S phase (arrows), G2 (G2), prophase (P) and metaphase (M). Scale bars, 10 µm. (**D** and **E**) Block of G2/M transition in Met-deficient livers shown by staining with phosphor-histone H3. Positive cells were counted in 10–20 randomly selected fields in *Met^wt/wt^;Alb-Cre^+/−^* (**D**) and *Met^f/f^;Alb-Cre^+/−^* (**E**) liver sections under confocal microscope and expressed as percent from a total of 600–2000 nuclei. Results shown are the means ± SE (n = 5−7 mice/group/time point).

To evaluate the effect of *c-met* deletion on cell cycle progression, we used 5-bromo-2′-deoxyuridine (BrdU) immunohistochemistry and mitotic frequency. A quantitative time course of BrdU incorporation showed that kinetics of DNA synthesis was conserved within the first 36 hr after PH but the peak level was significantly reduced in c-Met mutant livers (Figure 1A). However, despite normal exit from quiescence and progression through the G1/S phases, the first wave of mitoses was both remarkably decreased and delayed (Figure 1B). Immunostaining with additional markers of cell cycle progression confirmed almost complete lack of mitosis in c-Met mutant livers at this time point (Figure 1C). Eventually, c-Met deficient hepatocytes entered mitosis albeit at a significantly reduced frequency (Figure 1B). Thus, analysis of cell cycle progression indicates that lack of c-Met function in hepatocytes inhibited cell proliferation through a combination of an initial G2/M block followed by a decreased entry into next G1 phase.

### c-Met Deficient Hepatocytes Are Blocked in Early/Mid G2

To determine more precisely when c-Met activity is required for transition into mitosis, we examined histone H3 phosphorylation as a differential marker for G2 and M phase cells. Histone H3 phosphorylation on Serine 10 (H3-P) represents a key step in chromosome remodeling during cell division [13]. It begins during early G2 in pericentric foci well before chromosome condensation is microscopically evident and reaches maximal levels in metaphase chromosome [14]. Immunoflourescence detection and quantitative assessment of H3-P staining revealed that the number of cells displaying a punctuate pattern of staining progressively increased in mice of both genotypes within the fist 24–36 hr as cells were completing the S phase and entering G2 phase. However, *Met^fl/fl^;Alb-Cre^+/−^* hepatocytes did not acquire a strong genome-wide H3-P required for chromosome condensation and mitotic onset and did not advance into mitosis (Figure 1D and E). These data suggest that c-Met deficient hepatocytes were blocked in early/mid G2 phase due to failure of mitosis-specific phosphorylation events responsible for chromosome compaction.

### Deregulation of Genes Involved in G2/M Progression

**Figure 2:**
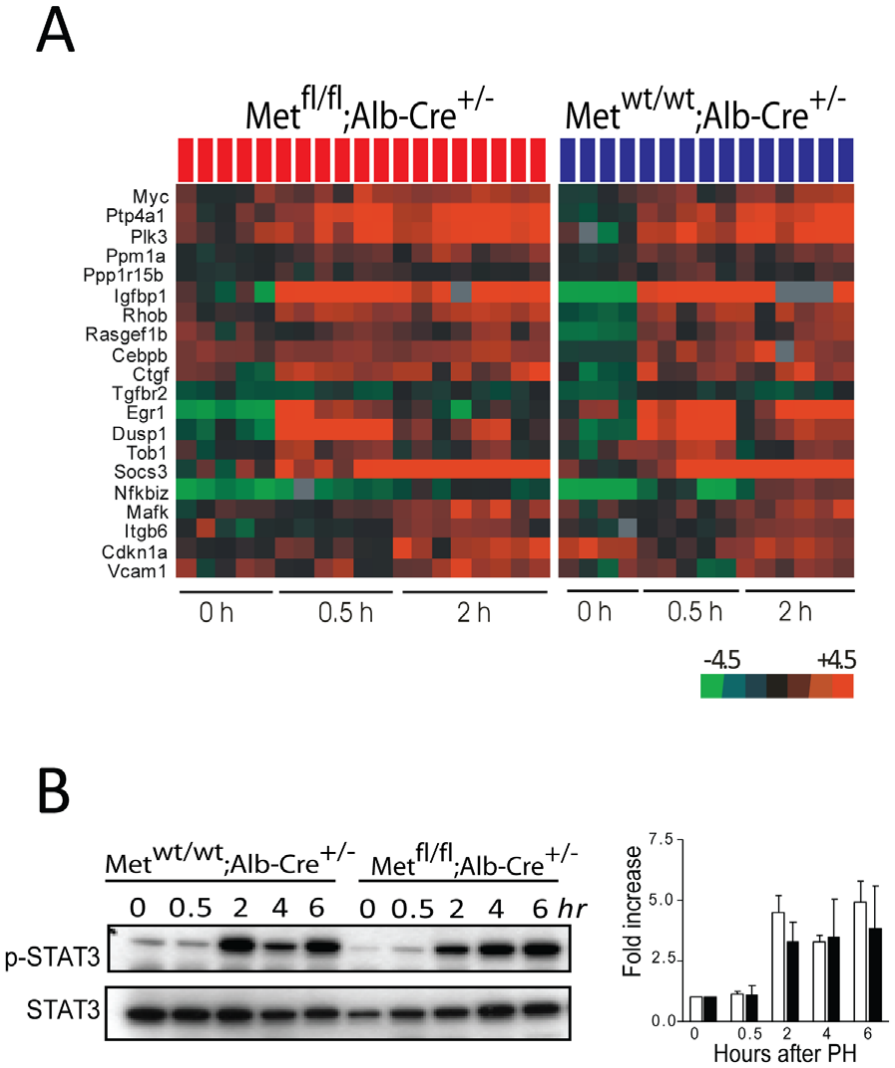
Induction of genes associated with priming phase in c-Met mutant livers after partial hepatectomy. (**A**) Heatmap depicts a subset of 20 genes that are commonly regulated in *Met^wt/wt^;Alb-Cre^+/−^* and *Met^fl/fl^;Alb-Cre^+/−^* mice within the first 2 hr of liver regeneration (1.5-fold, *P*<0.01 in at least 80% of arrays). (**B**) Representative western blots and quantification of total and phosphorylated Stat3 protein levels relative to β-actin. Results shown are the means ± SE of 3 experiments.

To address the molecular basis of the early G2 block, we next examined the transcriptional response to partial hepatectomy using gene expression profiling. RNA was isolated from liver samples at various time-points of liver regeneration including the initial or priming phase (0.5, 2 hr) which allows rapid activation of transcription factors promoting G_0_-G_1_ transition of normally quiescent hepatocytes [15], transition through G1 phase (12, 24 hr) and S/G2/M (36, 42, 48 hr) phases (Figure 1). Only genes with more than 1.5-fold expression differences between control and c-Met mutant mice in at least 80% of samples were included in the list of significant genes. In agreement with cell kinetics data (Figure 1A), the early priming events appeared to be unaffected by the loss of c-Met signaling in hepatocytes consistent with the previous work demonstrating that the massive changes in gene expression in the first four hours after 2/3 PH, while preparing the quiescent hepatocytes to enter cell cycle, cannot predict whether DNA replication may actually occur [15]. Overall, there were no significant variations in the early induction of several key transcription factors involved in the initiation of hepatocyte proliferation including *c-Jun*, *c-Fos* and *c-Myc* between *Met^wt/wt^;Alb-Cre^+/−^* and *Met^fl/fl^;Alb-Cre^+/−^* mice (Figure 2A and Figure S3). Also, both TNFα and IL6 responses appeared to be similar in the knockouts as judged by a comparable induction of the known feedback regulators of the major cytokine and growth factor signaling pathways such as *SOCS3*, *IGFR1*, *DUSP1* and *DUSP 6*. Accordingly, mice of both genotypes exhibited a high degree of Stat3 phosphorylation, an important downstream effector of IL6 signaling, which peaked at 4–6 hr after PH (Figure 2B). Nonetheless, a subset of immediate-early genes implicated in the control of cell cycle progression, such as *c-Fos* and *Egr1*, exhibited an impaired activation in c-Met mutant livers, particularly at the later time points of liver regeneration (Figure S3).

**Figure 3:**
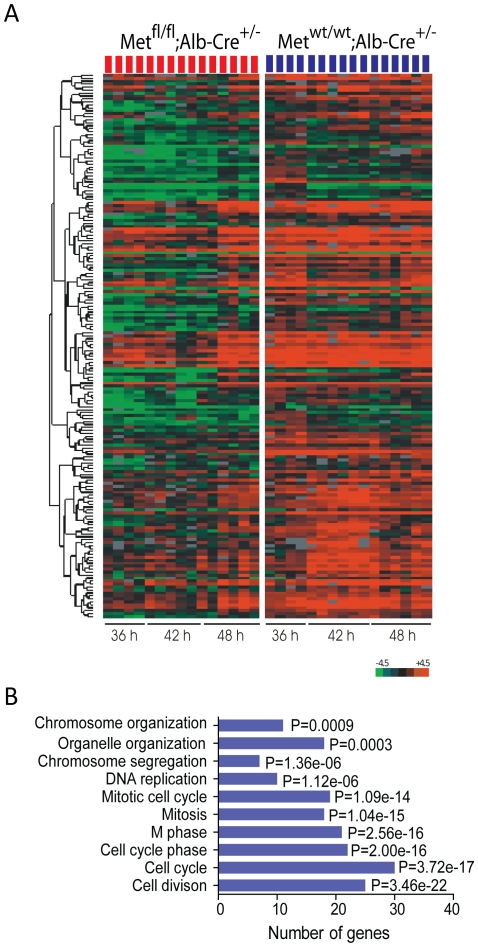
Downregulation of genes involved in G2/M progression in *Met^fl/fl^;Alb-Cre^+/−^* mice. (**A**) Expression profiles of 184 genes encoding G2/M regulators based on the Ingenuity Pathway Analysis. (**B**) The ten top-ranked Gene Ontology categories reflecting biological changes in Met mutant livers. Only the GO categories that were significantly underrepresented in Met livers are indicated together the number of genes altered in each category (X-axis).

However, the greatest differences in gene expression were detected at 36–48 hr when c-Met mutant cells were blocked in G2 phase. At this time, we identified 184 downregulated genes promoted by the absence of c-Met (*P≤*0.01) (Table S1, Figure 3A). Ingenuity Pathway Analysis of the down-regulated genes revealed that cell division, cell cycle, mitotic cell cycle, chromosome segregation and chromosome organization belonged to the top ten functions with the highest *P*-values (Figure 3B). Many of the identified cell cycle genes were found to be associated with G2/M cell cycle checkpoint/arrest including *Fen1*, *Cdc25b*, *Mbd4*, *Egr1*, *Ccnb1*, *Ccnb2*, *Plk4*, *Aurka*, and *Mad2L1*. Notably, c-Met inactivation affected expression of genes involved in chromosome congression, alignment and segregation (*Esco2*, *SMC2*, *Nuf2*). In addition, we found downregulation of several genes encoding kinetochore and centromere-associated proteins essential for faithful chromosome segregation during mitotic progression, such as *Ndc80*, *Cenp-e*, -*g*, -*h*, -*k*, -*i* and –*q*. Among those, *Cenpe* is a kinesin-like motor protein which accumulates in G2 phase just before the mitosis and required for the efficient recruitment of *BUBR1*, *MAD1* and *MAD2* to kinetochores [16]. Many of the over-represented genes with reduced expression are known to play important roles in spindle organization and biogenesis including *Ect2*, *ASPM*, *Nusap1*, *Kif18a* and *Stmn1*, *3*, and *4*. Furthermore, the expression of common markers of checkpoint/arrest involved in G1/S and S phase transition, such as *Ccne*, *Ccnd*, *Gas1*, and *Dbf4*, was also decreased. This was consistent with the observation that the number of S phase cells was reduced in c-Met mutant livers after 36 hr of liver regeneration as cells failed to transit into next S phase providing further support that c-Met deletion has a broad impact on cell cycle progression (Figure 1).

Finally, we used gene set enrichment analysis (GSEA) to compare the differentially expressed genes in *Met^fl/fl^;Alb-Cre^+/−^* regenerating livers against a gene set which was previously identified as G2/M phase regulated in synchronized HeLa cells [17]. This analysis revealed that more than a third of orthologous genes (48/132) belonging to the G2/M cluster as defined by HeLa expression data were significantly downregulated in *Met^fl/fl^;Alb-Cre^+/−^* mice at 36–48 hr (Figure S4). Thus, microarray profiling proves consistent with the cell kinetics data and emphasizes a previously unrecognized link between c-Met signaling and regulation of genes functionally involved in G2/M progression.

### G2 Block Correlates with the Lack of Erk1/2 Signaling

**Figure 4:**
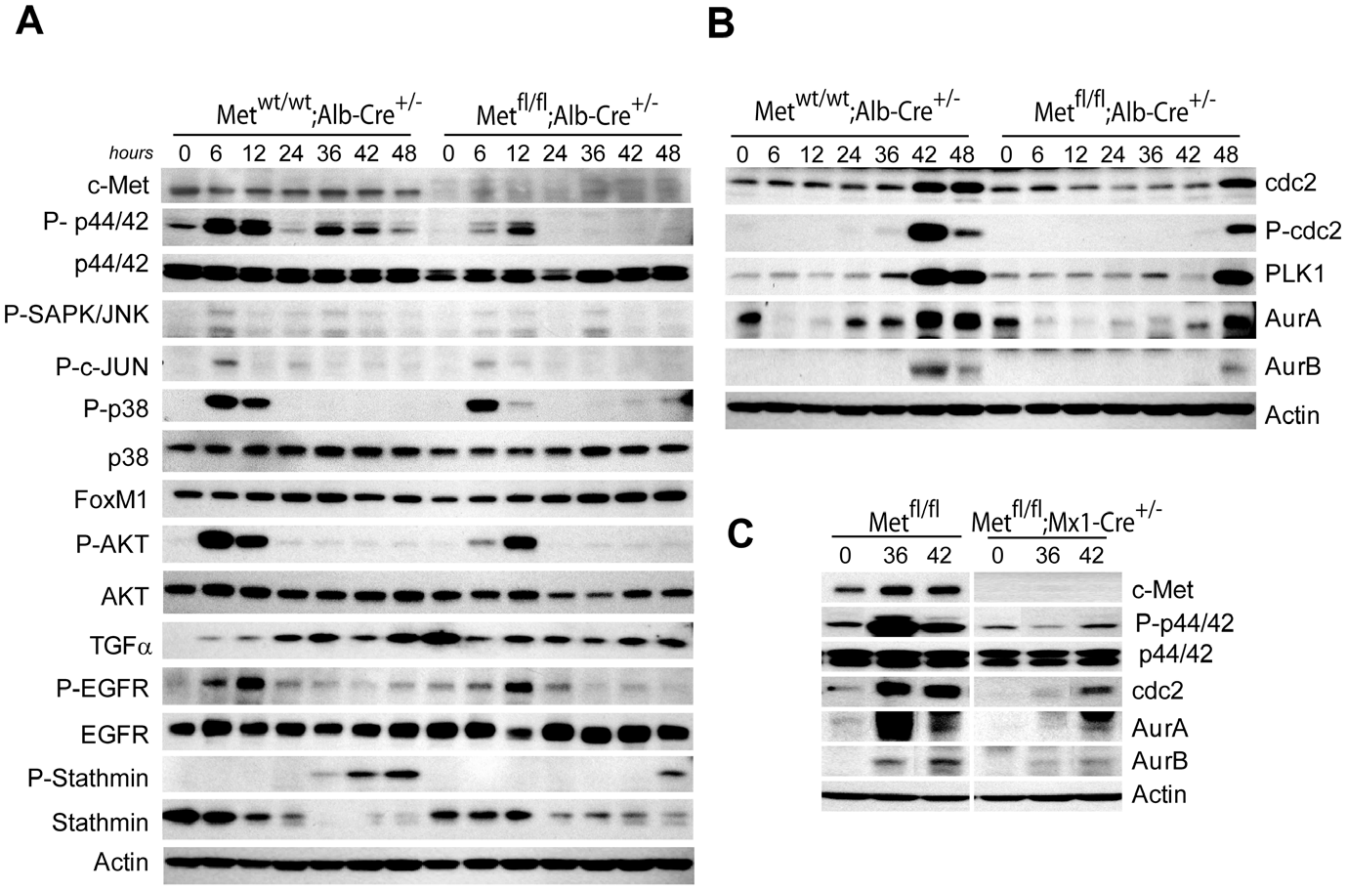
Downregulation of cell cycle regulated proteins in c-Met-deficient regenerating livers. (**A** and **B**) Whole cell lysates were prepared from *Met^wt/wt^;Alb-Cre^+/−^* and *Met^fl/fl^;Alb-Cre^+/−^* livers at the indicated times and probed by Western blotting using the indicated antibodies. (**C**) Western blotting of whole cell lysates prepared from *Met^fl/fl^* and *Met^fl/fl^;Mx1-Cre^+/−^* livers. Representative immunoblots are shown. Actin served as a loading control.

The critical role of c-Met in activation of the downstream mediator Erk1/2 during liver regeneration has been established using a liver-specific c-Met conditional knockout mouse model [7]. Therefore, the hepatocyte-specific c-Met conditional knockout mice were also examined for the defects in the Erk1/2 phosphorylation. As expected, c-Met protein was not detectable in whole cell lysates prepared from the mutant livers at any time point during liver regeneration (Figure 4A). Deletion of c-Met in hepatocytes did not affect the early phosphorylation of Erk1/2 at 6–12 hr following PH but it completely abolished the second peak between 36–48 hr coincidently with a defective progression through G2 phase of cell cycle (Figure 4A). Since *Met^fl/fl^;Alb-Cre^+/−^* hepatocytes express a kinase-inactive c-Met receptor which is irresponsive to HGF stimulation [8], these data indicate that c-Met signaling in hepatocytes is dispensable for initiation but required for sustaining a long-term Erk1/2 phosphorylation during liver regeneration.

Activation of other members of MAPK family, including a well known regulator of G2/M transition p38 MAPK, appeared to be normal. In addition, we observed only minimal if any changes in phosphorylation of Akt, another important downstream mediator of c-Met pathway. More significantly, there were no differences in the expression of TGFα or phosphorylation of EGFR receptor (Figure 4A) suggesting unique and important contribution of Met/Erk signaling in the induction of genes functionally involved in G2/M progression which is not compensated by other tyrosine kinase receptors.

### Ineffective Induction of Mitosis-Specific Kinases

Finally, we examined the protein levels of potential downstream targets of MAPK/Erk pathway which control various events during mitosis. We found a profound inhibition and delayed recruitment of several classes of mitosis-specific serine/threonine kinases including cdc2, Aurora A and B, and Plk1 (Figure 4B and Figure S5). Specifically, the depletion of Aurora B kinase, which associates with chromosomes during early mitosis as a part of chromosomal passenger complex [18], was consistent with a delayed histone H3 phosphorylation and failure of chromosome condensation. In addition, the protein levels of Mad2, a key component of the spindle checkpoint that inhibits anaphase promoting complex [19], were also reduced (Figure S5). To exclude the possibility that the lack of phosphorylation of mitotic kinases was due to a secondary effects caused by long-term adaptive changes in *Met^fl/fl^;Alb-Cre^+/−^* mice, we repeated the experiments using regenerating livers from *Met^fl/fl^;Mx1-Cre^+/−^* conditional knockout mice. The results showed that cdc2 and Aurora B expression were similarly repressed at 36–42 hr concordant with the lack of ERK1/2 activation (Figure 4C).

**Figure 5:**
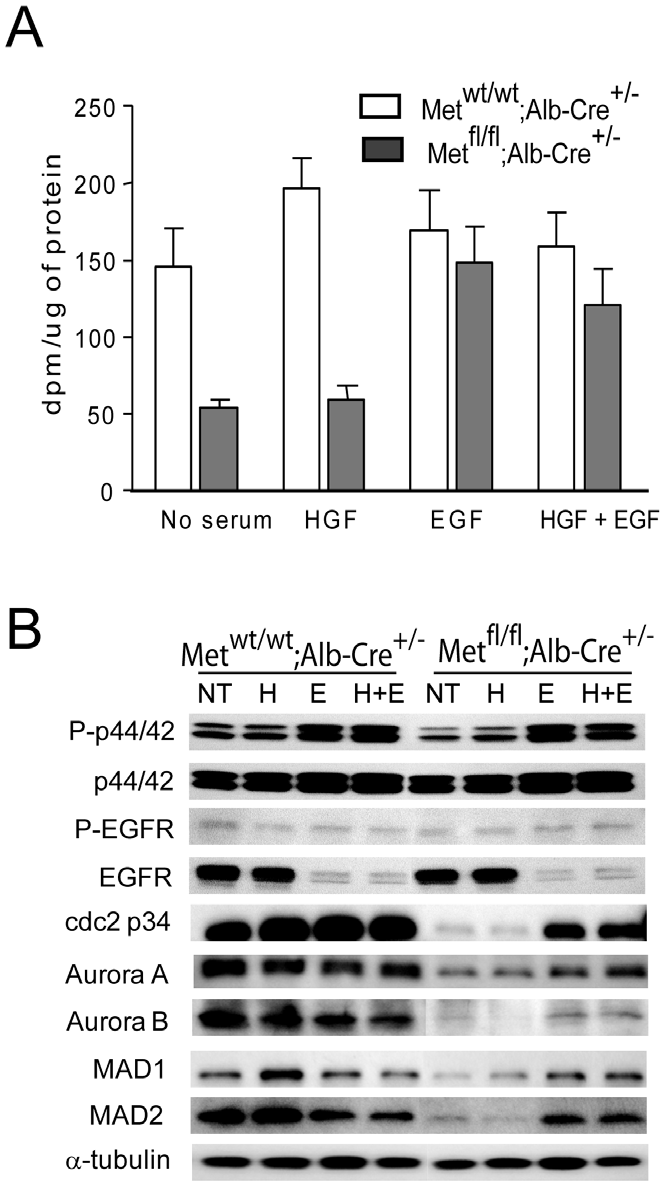
Reduced DNA replication and diminished expression of cell cycle proteins in c-Met deficient primary hepatocytes. (**A**) Hepatocytes were isolated from 2-month-old *Met^wt/wt^;Alb-Cre^+/−^* and *Met^fl/fl^;Alb-Cre^+/−^* livers and cultured in the presence of HGF (40 ng/ml), EGF (20 ng/ml) or HGF+EGF for 3 days. DNA replication was analyzed by ^3^H-Thymidine incorporation expressed as counts per minute per microgram of protein. Cells were exposed to ^3^H-Thymidin for 24 hr before harvesting. Shown are the means ± SE of duplicates from 4 independent experiments. (**B**) Western blot analysis of whole cell lysates with indicated antibodies. Cells were grown in the absence (NT) or presence of HGF (H), EGF (E), and both (H+E) for 3 days. The experiments were performed twice, and representative immunoblots are shown. α-tubulin served as a loading control.

We also used primary cultured hepatocytes to address the effect of c-Met deficiency on proliferation and intracellular signaling. The proliferation of primary hepatocytes in culture as well as expression of cell cycle regulators were correspondingly compromised in the absence of c-Met signaling (Figure 5A and B). Continuous EGF stimulation for 72 hr was able to increase Erk1/2 phosphorylation and restore both DNA synthesis and protein levels of cdc2, Aurora A, Aurora B, and Mad1and 2 albeit to a considerably lesser degree as compared to control hepatocytes, again underscoring a key contribution of c-Met to maintaining an adequate levels of cell proliferation.

## Discussion

Using regenerating mouse liver as a model of cell cycle progression, we investigated the role for c-Met in hepatocyte proliferation. We report that absence of c-Met signaling in hepatocytes results in G2/M cell cycle arrest along with the lack of Erk1/2 activation and downregulation of broad G2/M regulatory networks. These results define a new critical function for c-Met in G2/M transition and implicate Erk1/2 as a potential G2/M target of c-Met pathway.

Until now, it was generally accepted that c-Met plays a pivotal role in regulating onset of S phase. Our results support earlier work describing the importance of Met/Erk1/2 in mitogenic signaling during liver regeneration [7]. By employing a model deficient for *c-met* exclusively in hepatocytes, we found that *c-met* ablation affected neither early hepatic Erk1/2 activation nor induction of the initial round of DNA replication. However, it totally abrogated the second peak of Erk1/2 phosphorylation at 36–48 hr and delayed cell cycle progression from G2 to M phase. The decrease in mitoses was followed by a reduced hepatocyte entry into the next S phase indicating that c-Met controls cell cycle progression at G1/S and G2/M check points. Interestingly, the induction of immediate early genes *c-Fos* and *Egr-1* which can function as sensors for duration of Erk1/2 signaling [20] was reduced in c-Met mutant mice, particularly at the later time points of liver regeneration (Figure S3). Downregulation of *Egr1* may be of significance to c-Met phenotype since *Egr1* is reported to be a transcriptional target of *HGF*, and *Egr1*-null mice display a delayed mitotic progression through defective spindle assembly checkpoint [21].

A described block at the G2/M boundary contrasts with a defective exit from quiescence and a complete lack of Erk1/2 activation observed in liver-specific c-Met conditional knockout mice which lost c-Met mediated signaling in all cell types [7]. The differences in the dynamics of Erk1/2 phosphorylation between two c-Met conditional knockout models imply that alternative pathways may account for the early Erk1/2 activation and that c-Met signaling in hepatocytes is essential for sustaining long term Erk1/2 activation throughout liver regeneration. It was suggested that cross talk between two receptor tyrosine kinases, c-Met and EGFR, may influence each other's activities by affecting the common downstream signaling pathways [22]. However, a complete absence of EGFR in the liver was insufficient to suppress c-Met and Erk1/2 activation [23] consistent with c-Met being a major determinant of mitogen-activated protein kinase cascades in regenerating liver [7]. Instead, lack of EGFR signaling in *EGFR^fl/fl^;Mx1-Cre* mutant livers reduced the cyclin D expression and blocked G1/S transition due to the reduced NF-kB activation [23], thus emphasizing the requirement of simultaneous co-activation of parallel signaling pathways and coordinated cellular responses for full mitogenic signaling and efficient liver regeneration.

The role of MAPK/Erk pathway in proliferation of rodent hepatocytes is well known [24]–[26]. There is extensive evidence that Erk1/2 activation is a prerequisite for G1 to S entry [27]. However, the contribution of Erk1/2 signaling to G2/M progression is less defined and more controversial being either stimulatory or inhibitory depending on the cell type and kinetics or strength of Erk1/2 phosphorylation [28]–[30]. Our findings are in agreement with the results of other studies demonstrating delayed G2/M kinetics upon pharmacologic MAPK inhibition, siRNA-mediated Erk1/2 knockdown, or dominant-negative Mek1/2 [31]–[33]. Notably, c-Met deficient hepatocytes were incapable of generating a sustained Erk1/2 signaling and after a successful passage through the first S phase were blocked in early/mid G2 phase concomitantly with suppression of the core G2/M genes, such as *Egr1*, *Ccnb1*, *Ccnb2*, *Plks*, *Aurka*, and *Mad2L1* [19], [34]–[37]. Inhibition of Erk1/2 activation was also associated with reduced global histone H3 phosphorylation, essential for chromosome condensation [13]. Accordingly, microarray profiling of c-Met deficient regenerating livers revealed downregulation of genes known to control chromosome congression, alignment and segregation (*Esco2*, *CENPs, SMC2*, *Nuf1*, etc.) along with others implicated in spindle organization and biogenesis (*Ect2*, *ASPM*, *Nusap1*, and *Stmn1*, *3* and *4*). In addition, *Met^fl/fl^;Alb-Cre^+/−^* livers displayed downregulation of *Egr-1*, an immediate early growth response gene shown to be involved in the control of mitotic entry through the spindle assembly checkpoint [21]. Western blot analysis also revealed aberrant phosphorylation of stathmin, a microtubule-destabilizing protein [38] (Figure 4A), pointing to c-Met dependent structural changes as possible causes of delayed cell cycle progression. Indeed, cell division, mitotic cell cycle and chromosome segregation were among the top Gene Ontology terms (Figure 3B).

Most significantly, and consistent with lack of Erk1/2 phosphorylation, we found a profound down-regulation of all three classes of serine/threonine kinases that monitor various aspects of mitosis, including Cdk1, Plk1 and two members of Aurora family kinase, Aurora A and B (Figure 4). Among these, Cdk1 is the main kinase player during G2/M transition [39] which controls execution of a transcriptional program required for mitosis through phosphorylation of Plk1 and Aurora B and subsequent activation of FoxM1, a key transcription factor during G2/M transition [35], [40].

To compensate for the loss of Erk1/2 activation, hepatocyte cultures established from *Met^fl/fl^;Alb-Cre^+/−^* livers were grown in the continuous presence of EGF. A persistent EGF supplementation *in vitro* was able only partially rescue the effect of Erk1/2 downregulation in c-Met depleted hepatocytes and restore to some extent the levels of cdc2, Aurora A and B, and Mad2. These results highlight a dominant role for c-Met signaling in maintaining sufficient levels of Erk1/2 activation and suggest that loss of Met/Erk1/2 signaling cascade is responsible for the c-Met knockout phenotype. In conclusion, we report that c-Met function in hepatocytes is required for direct activation of Erk1/2 at the G2/M transition and coordinated control of G2/M transcriptional program.

## Materials and Methods

### Ethics Statement

All experimental procedures were approved by the NCI-Bethesda Animal Care and Use Committee (ACUC) under LEC-038. The NCI animal program meets or exceeds requirements of the Public Health Service Policy on Humane Care and Use of Animals and is fully accredited by AAALAC International.

### Mice

Hepatocyte specific c-Met conditional knockout (*Met^fl/fl^*;*Alb-Cre^+/−^*) and control (*Met^wt/wt^;Alb-Cre^+/−^*) mice were described previously. Since deletion of floxed DNA in hepatocytes is time-dependent [41], we used male mice at 8–10 weeks of age, when cre-recombination is complete (Figure S1A). Genotyping was done by PCR analysis on tail genomic DNA and specificity of the c-*met* deletion was verified by immunohistochemistry. Expression of c-Met was not detectable in *Met^fl/fl^;Alb-Cre^+/−^* hepatocytes and limited to nonparenchymal liver cells (Figure S1B).

To achieve systemic deletion of *c-met, Met^fl/fl^* mice [8] were bred with homozygous *Mx1-Cre* mice expressing *cre* under the control of type I IFN-inducible Mx1 promotor as described [7] to generate *Met^fl/fl^;Mx1-Cre^+/−^* mice. To activate Mx1 promotor, 8-week- old male mice received three i.p. injections of 300 µg poly (I:C) (Sigma-Aldrich) with a 2 day interval. Mx1-Cre-negative littermates (*Met^fl/fl^*) received equal amounts of poly (I:C) and served as controls. The partial hepatectomy was performed 3 days after the last injection. We used functional test to verify the efficient inactivation of the *c-met* gene after *Mx1-cre-*induced recombination. HGF mediated c-Met phosphorylation as well as downstream signaling via Erk1/2 and Akt were completely abolished in hepatocytes isolated from *c-Met^fl/fl^;Mx1-*Cre*^+/−^* mice (Figure S1C).

### Partial Hepatectomy (PH)

Mice were anesthetized with a mixture of isoflurane/oxygen, and right medial, left medial, and left lateral lobes were excised after separate ligations resulting in removal of ∼70% of the hepatic mass. A special care has been taken to preserve the gallbladder. At least 5 animals were used for each time point. Livers were harvested, weighed, and processed for subsequent analysis. Preoperative and regenerated liver weight was expressed as liver-to-body-weight ratio and used to calculate the relative growth of residual liver lobes as the regenerated/preoperative liver weight X 100%. Preoperative liver weight in untreated control and mutant mice was 4.5 and 4.1 (*P*<0.05), respectively, reflecting small albeit persistent differences in liver mass (Figure S1E).

### Analysis of Liver Function

Blood samples were obtained from orbital venous plexus of 3 mice per time point under isoflurane anesthesia before sacrifice. Serum levels of alanine aminotransferase (ALT), albumin, and triglyceride, were measured using a Reflovet Plus bench top analyzer (Roche) following manufacturer's instructions.

### BrdU Labeling and Immunohistochemistry

Mice received i.p. injections of 150 µg/g 5-bromo-2′-deoxyuridine (BrdU) (Roche Applied Science) 2 hr before sacrifice. Livers were fixed in alcoholic-formalin for 4–6 hr, 5-µm paraffin sections were prepared and stained with ant-BrdU (1∶100; Becton Dickinson), anti-phosphor-histone H3 Ser10 (1∶200; Cell Signaling) or PCNA clone PC10 (1∶200; DAKO Cytomation) and examined with a Zeiss 510 confocal laser scanning microscope (Carl Zeiss, Germany).

### Western Blotting

Whole cell lysates (100 µg) were prepared from frozen tissues using T-PER Tissue Extraction Buffer (Pierce) containing Complete Protease Inhibitor Cocktail (Roche), separated by SDS-PAGE and transferred onto Invitrolon PVDF (Invitrogen). Membranes were blocked in 5% non-fat milk in Tris-buffered saline containing 0.1% Tween 20 for 1 h and probed with specific antibodies listed in Table S2. Immune complexes were detected using the enhanced chemiluminescence system (Pierce).

### Primary Hepatocyte Culture and [^3^H]Thymidine Incorporation

Hepatocytes were isolated from 2-month-old mice using a two-step collagenase perfusion [8]. The rate of DNA synthesis was measured by [methyl-3H]thymidine incorporation as described [26]. Cells were incubated with 1 µCi of [methyl-3H]thymidine (5 Ci/mmol) for 24 hr before cell harvesting, washed twice with PBS, scrapped off the culture dish and divided for analysis of protein content and [3H]thymidine counting using a MicroBeta TriLux scintillation counter (Perkin Elmer).

### Microarray Experiments and Data Analysis

Microarrays containing 21,997 long (65-mer) oligonucleotides representing 19,740 unique genes were printed in the Laboratory of Molecular Technology (National Cancer Institute). Total RNA was isolated from frozen liver samples using TRIZOL (Invitrogen) following the manufacture's recommendation. Sample preparation and array hybridization were performed as described [42]. Briefly, 20 µg of RNA were converted to cDNA, labeled with Cy-3 or Cy-5 dye and hybridized together with common reference RNA in a dye-swap design. The common reference RNA was pooled from ten wild type B6/129 strain mouse livers. Gene expression values defined as a sample-per-reference ratio [42] were normalized by Loess and quantile methods across all samples [43], [44] and log_2_ transformed. The microarray datasets have been deposited to Gene Expression Omnibus database (http://www.ncbi.nlm.nih.gov/geo, accession number GSE17609).

The cell cycle progression in the regenerative livers is only a partially synchronized process. Therefore, to identify the biologically relevant gene expression changes, samples at early (30 min-2 hr) and late (36–42–48 hr) time points were pooled together and analyzed as single data sets to minimize the intrinsic variations between different animals while increasing the statistical power of comparison. Differentially expressed genes were selected across the selected time points (early versus late) using bootstrap *t*-test [45] with 6,000 repetitions (*P≤*0.01) and at least 1.5 fold expression changes between *Met^fl/fl^;Alb-Cre^+/−^* and *Met^wt/wt^;Alb-Cre^+/−^* mice. All selected genes satisfied the criteria for false discovery rate of less than 0.1 which was calculated using “fdrtool” R version 2.11.0 as described [46]. RT-qPCR was performed for selected targets using SYBR Green Master-Mix (Bio-Rad) to validate the microarray results.

Data mining was performed using Ingenuity Pathway Analysis tool (Ingenuity Systems Inc.). The significance of each network, function, and pathway was determined by the scoring system provided by Ingenuity. The differentially expressed genes were compared with the genes which were previously identified as G2/M phase regulated in synchronized HeLa cells [17]. Orthologous genes between mouse and human microarrays were selected using HomoloGene database of National Center for Biotechnology Information (NCBI). The gene set enrichment analysis (GSEA) was performed using GSEA v2.06 (Broad Institute, MIT).

## Supporting Information

Figure S1 Selective disruption of c-Met in hepatocytes does not affect postnatal liver growth. (A) PCR analysis of floxed *c-met* allele (*Met^fl/fl^*). Genomic DNA was isolated from livers at different age. (B) Indirect immunofluorescence staining of liver sections with c-Met antibody. Nuclear counterstaining was performed with DAPI. Expression of c-Met was not detectable in *Met^fl/fl^;Alb-Cre^+/−^* hepatocytes and limited to nonparenchymal liver cells (arrows). Scale bar, 10 µM. (C) Western blots showing phosphorylation status of c-Met, p42/p44 MAPK and Akt upon HGF stimulation in pimary hepatocytes. Cells were isolated from adult *Met^fl/fl^* and *Met^fl/fl^;Mx1-Cre^+/−^* livers using a two-step collagen perfusion followed by isodensity centrifugation. After overnight incubation, cells were synchronized by serum deprivation for 2 hrs and treated with 50 ng/ml of rhHGF (PeproTech) for 5–30 min. Note that HGF mediated c-Met phosphorylation as well as downstream signaling via Erk1/2 and Akt were completely abolished in *c-Met^fl/fl^;Mx1-Cre^+/−^*. (D) Representative FACS histograms of DNA content and ploidy distribution in *Met^wt/wt^;Alb-Cre^+/−^* and *Met^fl/fl^;Alb-Cre^+/−^* livers at 2 months of age. Freshly isolated hepatocytes were stained with propidium iodine using the Cell Cycle Test DNA Reagent Kit (Becton-Dickinson, San Jose, CA). Nuclei DNA content was measured using a Becton-Dickinson FACScan flow cytometer and Cell-Quest Sofware. 20,000 events were collected. Results shown are the means ± SE (n = 3 mice per group). (E) Liver/body weight ratios and blood biochemistry. Serum was obtained from *Met^wt/wt^;Alb-Cre^+/−^* and *Met^fl/fl^;Alb-Cre^+/−^* at 2 month of age. Results shown are the means ± SE (n = 3 mice per group). Asterisk indicates statistical significance assessed by Student's t test (*P*<0.05). M, Marker; AST, Aspartate aminotrasferase, ALT, Alanine aminotrasferase.
(2.00 MB PDF)

Figure S2 Loss of Met delays recovery of liver mass and function after partial hepatectomy. (A) Serum levels of aspartate aminotrasferase (AST) were indistinguishable in control and Met-deficient mice indicating a similar extent of hepatic injury. (B) Slower recovery of liver weight and reduced levels of albumin (C) and triglyceride (D) in Met-deficient livers. Data are shown as the means ± SE (n = 3−5/group per time point). Asterisks indicate statistical significance assessed by Student's *t* test (*P* is less than at least 0.05).
(0.33 MB PDF)

Figure S3 Temporal profiles of mRNA levels of selected genes during liver regeneration. The results from microarray and the corresponding RT-qPCR analyses are shown in left and right panels, respectively. Oligonucleotide primers were designed using Primer3 v.0.4.0 (http://frodo.wi.mit.edu/primer3/). The amplification protocol was as follows: 95°C for 3 min, followed by 40 cycles of 95°C for 15 seconds and 1 minute at 60°C, completed by a dissociation curve to identify false positive amplicons. The relative expression level of each gene was normalized to the corresponding levels at 0 hr and calculated using the formula 2^(−ΔΔCt)^. GAPDH and 18s RNAs were used as endogenous reference. The data are presented as the means ± SD (n = 3). Asterisks indicate statistical significance assessed by Student's *t* test (*P* is less than at least 0.05).
(0.40 MB PDF)

Figure S4 Gene set enrichment analysis (GSEA). This analysis was performed to compare gene expression data of regenerating mouse livers with a gene set identified as G2/M phase regulated in synchronized HeLa cells {Whitfield, 2002 #17}. To explore the enrichment of G2/M associated genes, we selected orthologous genes between human and mouse microarrays using HomoloGene database of National Center for Biotechnology Information (NCBI). A total of 132 orthologous genes were present at the G2/M stage. (A) Enrichment of the G2/M gene set in *Met^fl/fl^;Alb-Cre^+/−^* phenotype (normalized enrichment score, NES  = 4.23, *P* value <0.0001). (B) The expression values of 48 out of 132 orthologous genes involved in G2/M progression were significantly downregulated in *Met^fl/fl^;Alb-Cre^+/−^* mice at 36–48 hr.
(0.28 MB PDF)

Figure S5 Western blot analysis of cell cycle-associated genes using nuclear extracts from timed liver samples after partial hepatectomy. Samples were probed by Western blotting using the indicated antibodies.
(0.67 MB PDF)

Table S1 List of genes differentially expressed in *Met^fl/fl^;Alb-Cre^+/−^* regenerating livers at 36–48 hr (>1.5 fold changes, *P*<0.01).
(0.20 MB DOC)

Table S2 List of antibodies.
(0.05 MB DOC)
