# Supplementary figures and images for: Loss of c-Met Disrupts Gene Expression Program Required for G2/M Progression during Liver Regeneration in Mice
Source: PLoS One. 2010 Sep 16;5(9):e12739. doi: 10.1371/journal.pone.0012739 (PMC2940888; doi:10.1371/journal.pone.0012739)

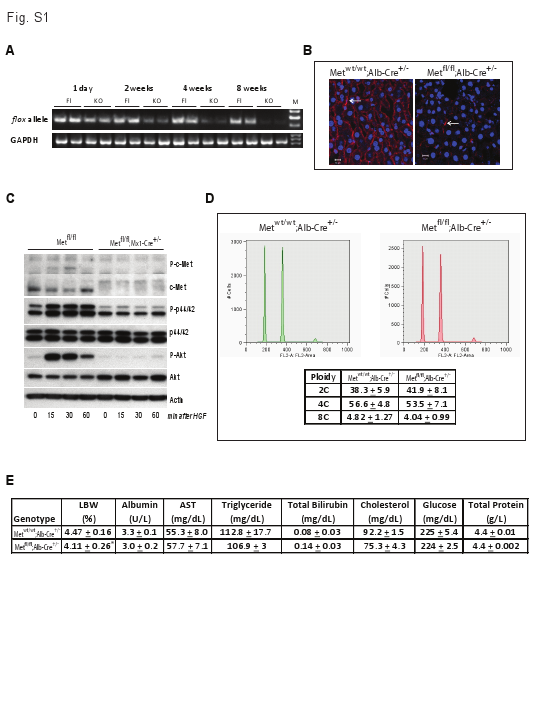

Supplement: Figure S1 — Selective disruption of c-Met in hepatocytes does not affect postnatal liver growth. (A) PCR analysis of floxed c-met allele (Metfl/fl). Genomic DNA was isolated from livers at different age. (B) Indirect immunofluorescence staining of liver sections with c-Met antibody. Nuclear counterstaining was performed with DAPI. Expression of c-Met was not detectable in Metfl/fl;Alb-Cre+/− hepatocytes and limited to nonparenchymal liver cells (arrows). Scale bar, 10 µM. (C) Western blots showing phosphorylation status of c-Met, p42/p44 MAPK and Akt upon HGF stimulation in pimary hepatocytes. Cells were isolated from adult Metfl/fl and Metfl/fl;Mx1-Cre+/− livers using a two-step collagen perfusion followed by isodensity centrifugation. After overnight incubation, cells were synchronized by serum deprivation for 2 hrs and treated with 50 ng/ml of rhHGF (PeproTech) for 5–30 min. Note that HGF mediated c-Met phosphorylation as well as downstream signaling via Erk1/2 and Akt were completely abolished in c-Metfl/fl;Mx1-Cre+/−. (D) Representative FACS histograms of DNA content and ploidy distribution in Metwt/wt;Alb-Cre+/− and Metfl/fl;Alb-Cre+/− livers at 2 months of age. Freshly isolated hepatocytes were stained with propidium iodine using the Cell Cycle Test DNA Reagent Kit (Becton-Dickinson, San Jose, CA). Nuclei DNA content was measured using a Becton-Dickinson FACScan flow cytometer and Cell-Quest Sofware. 20,000 events were collected. Results shown are the means ± SE (n = 3 mice per group). (E) Liver/body weight ratios and blood biochemistry. Serum was obtained from Metwt/wt;Alb-Cre+/− and Metfl/fl;Alb-Cre+/− at 2 month of age. Results shown are the means ± SE (n = 3 mice per group). Asterisk indicates statistical significance assessed by Student's t test (P<0.05). M, Marker; AST, Aspartate aminotrasferase, ALT, Alanine aminotrasferase. (2.00 MB PDF) [file pone.0012739.s001.tif]

Fig. S2

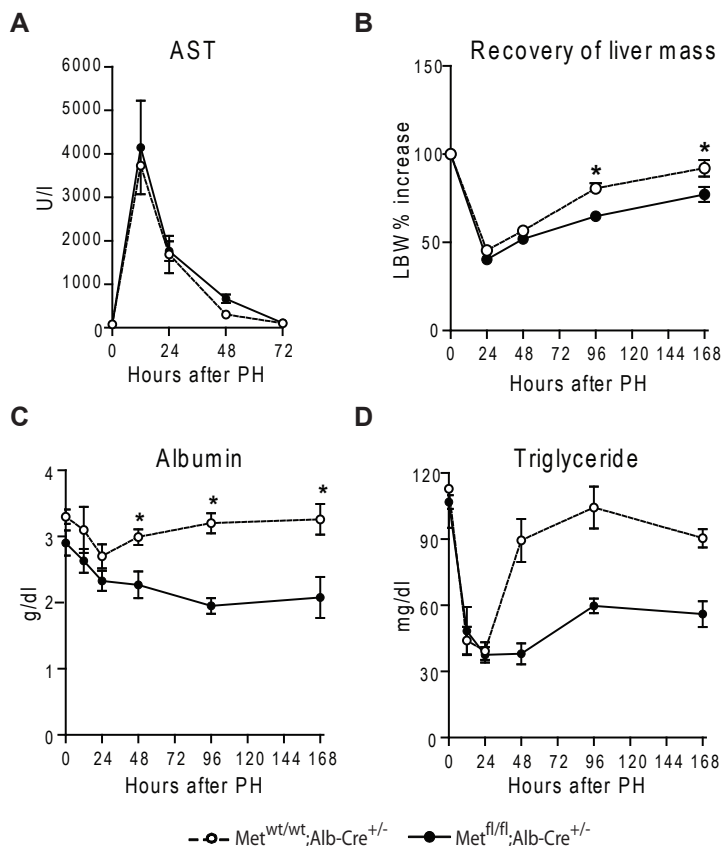

Supplement: Figure S2 — Loss of Met delays recovery of liver mass and function after partial hepatectomy. (A) Serum levels of aspartate aminotrasferase (AST) were indistinguishable in control and Met-deficient mice indicating a similar extent of hepatic injury. (B) Slower recovery of liver weight and reduced levels of albumin (C) and triglyceride (D) in Met-deficient livers. Data are shown as the means ± SE (n = 3−5/group per time point). Asterisks indicate statistical significance assessed by Student's t test (P is less than at least 0.05). (0.33 MB PDF) [file pone.0012739.s002.pdf]

Fig. S3

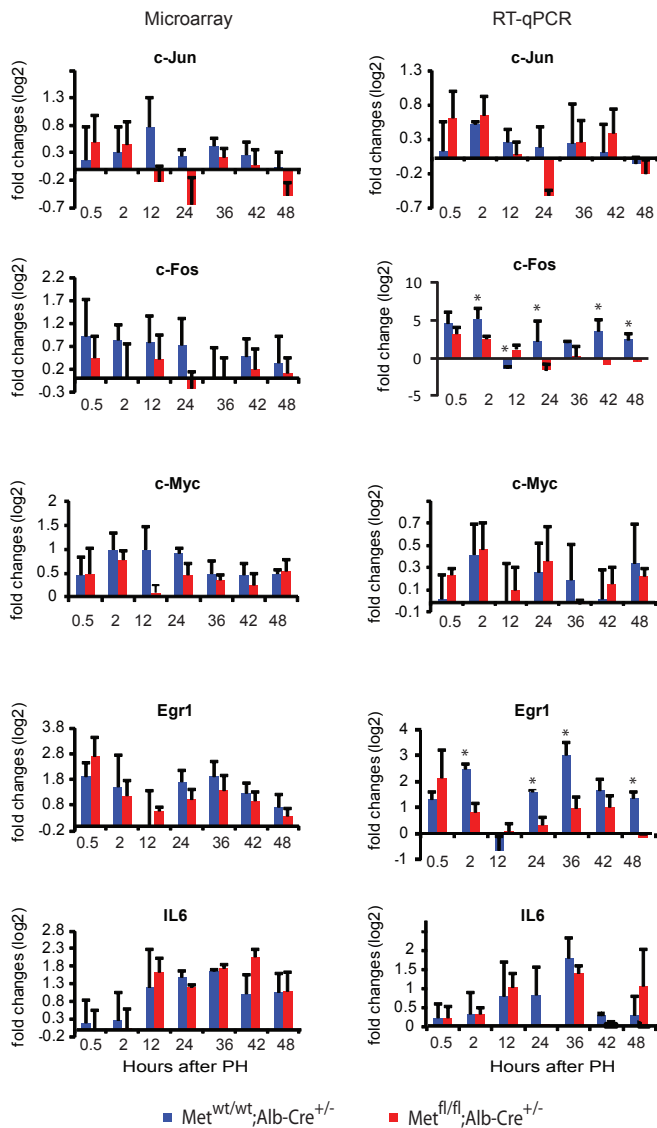

Supplement: Figure S3 — Temporal profiles of mRNA levels of selected genes during liver regeneration. The results from microarray and the corresponding RT-qPCR analyses are shown in left and right panels, respectively. Oligonucleotide primers were designed using Primer3 v.0.4.0 (http://frodo.wi.mit.edu/primer3/). The amplification protocol was as follows: 95°C for 3 min, followed by 40 cycles of 95°C for 15 seconds and 1 minute at 60°C, completed by a dissociation curve to identify false positive amplicons. The relative expression level of each gene was normalized to the corresponding levels at 0 hr and calculated using the formula 2(−ΔΔCt). GAPDH and 18s RNAs were used as endogenous reference. The data are presented as the means ± SD (n = 3). Asterisks indicate statistical significance assessed by Student's t test (P is less than at least 0.05). (0.40 MB PDF) [file pone.0012739.s003.pdf]

Fig. S4

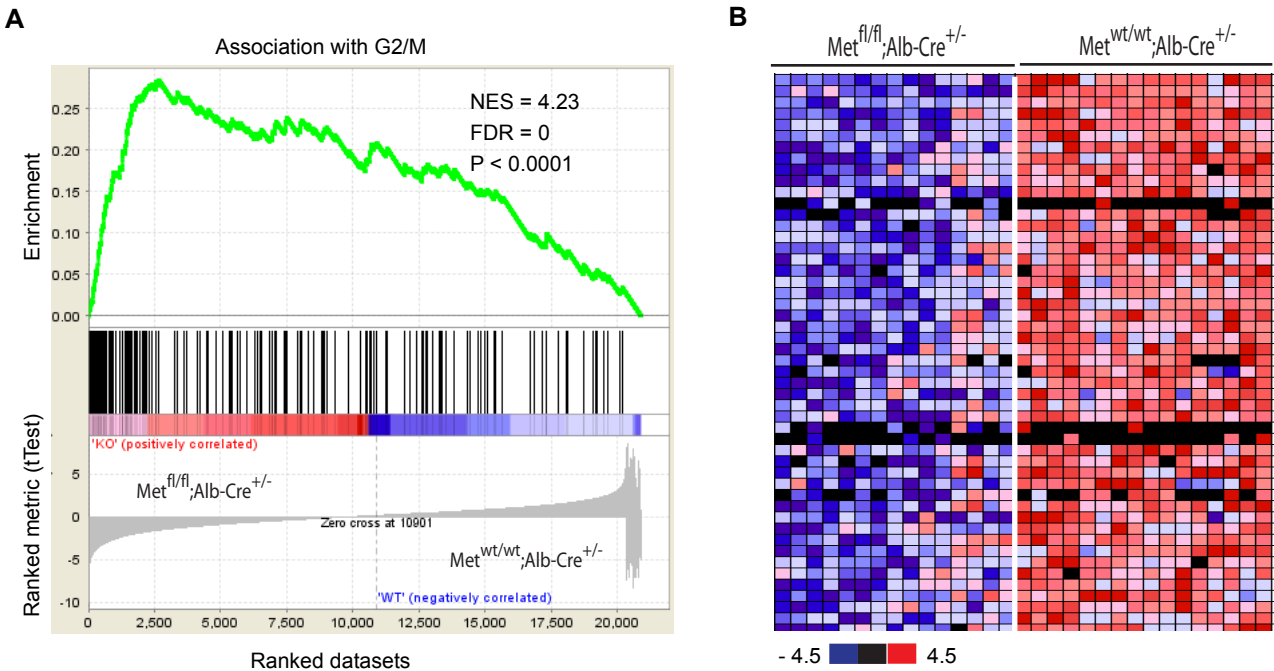

Supplement: Figure S4 — Gene set enrichment analysis (GSEA). This analysis was performed to compare gene expression data of regenerating mouse livers with a gene set identified as G2/M phase regulated in synchronized HeLa cells {Whitfield, 2002 #17}. To explore the enrichment of G2/M associated genes, we selected orthologous genes between human and mouse microarrays using HomoloGene database of National Center for Biotechnology Information (NCBI). A total of 132 orthologous genes were present at the G2/M stage. (A) Enrichment of the G2/M gene set in Metfl/fl;Alb-Cre+/− phenotype (normalized enrichment score, NES = 4.23, P value <0.0001). (B) The expression values of 48 out of 132 orthologous genes involved in G2/M progression were significantly downregulated in Metfl/fl;Alb-Cre+/− mice at 36–48 hr. (0.28 MB PDF) [file pone.0012739.s004.pdf]

Fig. S5

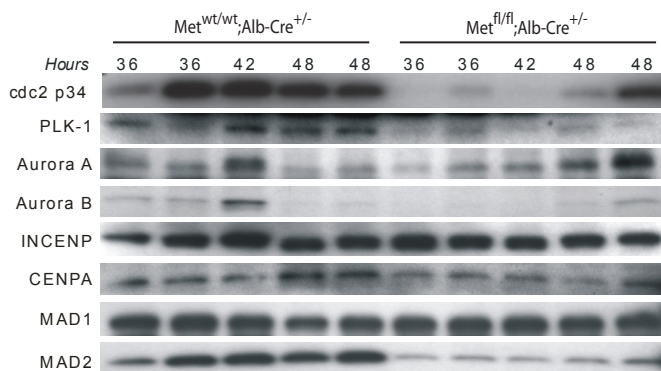

Supplement: Figure S5 — Western blot analysis of cell cycle-associated genes using nuclear extracts from timed liver samples after partial hepatectomy. Samples were probed by Western blotting using the indicated antibodies. (0.67 MB PDF) [file pone.0012739.s005.pdf]
