# Supplementary material for: Loss of c-Met Disrupts Gene Expression Program Required for G2/M Progression during Liver Regeneration in Mice
Source: PLoS One. 2010 Sep 16;5(9):e12739. doi: 10.1371/journal.pone.0012739 (PMC2940888; doi:10.1371/journal.pone.0012739)
Supplement: Table S1 — List of genes differentially expressed in Metfl/fl;Alb-Cre+/− regenerating livers at 36–48 hr (>1.5 fold changes, P<0.01). (0.20 MB DOC) [file pone.0012739.s006.doc]

**Table S1.** List of genes differentially expressed in Met^fl/fl^;Alb-Cre^+/-^ regenerating livers at

36-48 hr (*P < 0.01* and *> 1.5* fold changes)

| **Gene**  **Symbol** | **UniGene ID** | **GenBank ID** | **Bootstrap**  **P-value** | **Log(fold changes)** |
| --- | --- | --- | --- | --- |
| Amy1 | Mm.439727 | 11722 | 0 | -0.62826082 |
| Birc5 | Mm.8552 | 11799 | 0.0013333 | -0.752706148 |
| Blm | Mm.12932 | 12144 | 0 | -0.595805546 |
| Bub1b | Mm.29133 | 12236 | 0.0041667 | -0.76650241 |
| Calm2 | Mm.329243 | 12314 | 0 | -0.849325276 |
| Aspm | Mm.168523 | 12316 | 0.0016667 | -0.935169311 |
| Cav2 | Mm.396075 | 12390 | 0 | -0.813723383 |
| Cbx3 | Mm.393602 | 12417 | 0.0001667 | -0.59350019 |
| Ccnb2 | Mm.22592 | 12442 | 0.0011667 | -1.233607467 |
| Ccnd1 | Mm.273049 | 12443 | 0 | -0.862984944 |
| Ccne2 | Mm.35867 | 12448 | 0.0003333 | -1.104016195 |
| Ces1 | Mm.22720 | 12623 | 0.001 | -0.703681679 |
| Cldn1 | Mm.289441 | 12737 | 0.001 | -0.663929148 |
| Cxadr | Mm.66222 | 13052 | 0.0015 | -0.608688717 |
| Cyp2a4 | Mm.154643 | 13086 | 0.0008333 | -0.684171122 |
| Cyp2c29 | Mm.20764 | 13095 | 0.0001667 | -0.895588978 |
| Cyp2c37 | Mm.38963 | 13096 | 0.001 | -0.799317862 |
| Slc26a2 | Mm.24803 | 13521 | 0.001 | -0.615493388 |
| Egr1 | Mm.181959 | 13653 | 0 | -1.970811974 |
| Fen1 | Mm.2952 | 14156 | 0 | -1.131934097 |
| Smc2 | Mm.2999 | 14211 | 0.0011667 | -0.889856234 |
| Gm2a | Mm.287807 | 14667 | 0.0001667 | -0.818441916 |
| Hao1 | Mm.26634 | 15112 | 0 | -0.704746987 |
| Hells | Mm.57223 | 15201 | 0.0048333 | -0.957916169 |
| Foxq1 | Mm.44235 | 15220 | 0 | -1.119629429 |
| Hmgn2 | Mm.319660 | 15331 | 0 | -0.900248905 |
| Hrsp12 | Mm.143977 | 15473 | 0 | -0.657933761 |
| Hsd11b1 | Mm.28328 | 15483 | 0.0013333 | -0.725507069 |
| Hsd3b4 | Mm.475664 | 15495 | 0.0001667 | -1.255990708 |
| Hsd3b5 | Mm.17910 | 15496 | 0.0038333 | -0.801952314 |
| Fabp5 | Mm.741 | 16592 | 0.001 | -0.820028 |
| Kpna2 | Mm.12508 | 16647 | 0.0001667 | -0.62582351 |
| Stmn1 | Mm.378957 | 16765 | 0.0003333 | -0.9777618 |
| Mbd4 | Mm.259308 | 17193 | 0.0006667 | -1.075205472 |
| Mbl2 | Mm.30045 | 17195 | 0.002 | -0.835506426 |
| Mcm5 | Mm.5048 | 17218 | 0.0016667 | -0.718490208 |
| Mcm6 | Mm.4933 | 17219 | 0.0016667 | -0.705427148 |
| Rdh11 | Mm.291799 | 17252 | 0.0026667 | -0.631118822 |
| Mthfd2 | Mm.443 | 17768 | 0.0023333 | -0.642461971 |
| Mtnr1a | Mm.5133 | 17773 | 0.0001667 | -1.017935436 |
| Myef2 | Mm.18535 | 17876 | 0.0018333 | -0.653111522 |
| Pcna | Mm.7141 | 18538 | 0.0036667 | -0.616594843 |
| Enpp2 | Mm.250256 | 18606 | 0 | -1.00273216 |
| Prim1 | Mm.2903 | 19075 | 0 | -0.651675777 |
| Rad21 | Mm.182628 | 19357 | 0 | -0.657833468 |
| Rad51 | Mm.330492 | 19361 | 0.0026667 | -0.672658179 |
| Raet1b | Mm.458004 | 19369 | 0 | -0.699811552 |
| Rbp1 | Mm.279741 | 19659 | 0 | -1.26665115 |
| Rpa2 | Mm.2870 | 19891 | 0.0003333 | -0.637437986 |
| Rpl7 | Mm.379004 | 19989 | 0.0001667 | -0.588999667 |
| Stmn3 | Mm.2319 | 20262 | 0.0065 | -0.826075077 |
| Ccl9 | Mm.416125 | 20308 | 0.0005 | -0.740598214 |
| Shcbp1 | Mm.37801 | 20419 | 0.0025 | -0.94757621 |
| Slc25a17 | Mm.222536 | 20524 | 0 | -0.796457523 |
| Tmpo | Mm.159684 | 21917 | 0.0036667 | -0.594971697 |
| Vnn1 | Mm.27154 | 22361 | 0.0046667 | -0.908053725 |
| Cenph | Mm.273502 | 26886 | 0.0045 | -0.609343811 |
| Dbf4 | Mm.292470 | 27214 | 0.0001667 | -1.0139188 |
| Hsd17b6 | Mm.26719 | 27400 | 0.0003333 | -0.743191145 |
| Slco1a1 | Mm.103665 | 28248 | 0 | -1.260748984 |
| Slco1a4 | Mm.458583 | 28250 | 0.0003333 | -0.851088351 |
| Gtse1 | Mm.20858 | 29870 | 0.0045 | -0.612790053 |
| Angptl3 | Mm.28341 | 30924 | 0.0056667 | -0.645074174 |
| H2afz | Mm.117541 | 51788 | 0.0001667 | -0.923651137 |
| Echdc1 | Mm.28930 | 52665 | 0.0001667 | -0.633773466 |
| Ppm1d | Mm.45609 | 53892 | 0 | -0.590408902 |
| Cks1b | Mm.3049 | 54124 | 0 | -0.999053497 |
| Mad2l1 | Mm.290830 | 56150 | 0 | -1.141848857 |
| Ttrap | Mm.427162 | 56196 | 0 | -0.603310332 |
| Sult1b1 | Mm.23502 | 56362 | 0.0001667 | -0.662011103 |
| Stmn4 | Mm.35474 | 56471 | 0.0035 | -0.849113575 |
| Raet1d | Mm.458004 | 56554 | 0 | -0.641682825 |
| Accn5 | Mm.88839 | 58170 | 0 | -0.967668511 |
| Cenpk | Mm.281498 | 60411 | 0.0005 | -0.830270136 |
| Fignl1 | Mm.236114 | 60530 | 0.005 | -0.810441364 |
| Keg1 | Mm.218561 | 64697 | 0.0013333 | -0.868941978 |
| Ap3m2 | Mm.390023 | 64933 | 0.0046667 | -0.658027509 |
| Fkbp11 | Mm.30729 | 66120 | 0.0001667 | -0.618199534 |
| Cks2 | Mm.222228 | 66197 | 0 | -0.836631294 |
| 2310007A19Rik | Mm.46405 | 66353 | 0 | -0.586599922 |
| Spc25 | Mm.272969 | 66442 | 0.0006667 | -0.874465933 |
| 2610039C10Rik | Mm.180363 | 66578 | 0.0001667 | -0.664125876 |
| Dnajc10 | Mm.21762 | 66861 | 0 | -0.614872563 |
| Asf1b | Mm.29680 | 66929 | 0.0061667 | -0.638976836 |
| Ndc80 | Mm.225956 | 67052 | 0.0033333 | -0.738823251 |
| Anubl1 | Mm.272772 | 67492 | 0 | -1.099601396 |
| Stx17 | Mm.171334 | 67727 | 0 | -0.609331257 |
| Cdca5 | Mm.23526 | 67849 | 0.0008333 | -0.60453532 |
| Tubb6 | Mm.181860 | 67951 | 0.0001667 | -0.720308777 |
| Ift80 | Mm.389451 | 68259 | 0 | -0.692416639 |
| Nrn1 | Mm.232930 | 68404 | 0.0001667 | -0.7168508 |
| Sgol2 | Mm.32800 | 68549 | 0.0008333 | -0.701796611 |
| 1110020G09Rik | Mm.244226 | 68646 | 0.0006667 | -0.700404486 |
| Dnaic1 | Mm.79127 | 68922 | 0 | -0.611311632 |
| 1700001L05Rik | Mm.66794 | 69291 | 0.0001667 | -0.889903882 |
| 2310057J16Rik | Mm.390010 | 69697 | 0 | -0.62650469 |
| Fyttd1 | Mm.12831 | 69823 | 0.0003333 | -0.708510546 |
| 2610002D18Rik | Mm.141592 | 69885 | 0.0003333 | -0.954740335 |
| 2810408I11Rik | | 69941 | 0.002 | -0.638830125 |
| Rdh20 | Mm.158320 | 70061 | 0 | -0.697477416 |
| Smc4 | Mm.206841 | 70099 | 0.0001667 | -1.047591783 |
| Nup205 | Mm.261208 | 70699 | 0 | -0.600955672 |
| Exod1 | Mm.274160 | 71151 | 0 | -0.770399326 |
| Mlf1ip | Mm.22108 | 71876 | 0.004 | -0.626881524 |
| Esco2 | Mm.249280 | 71988 | 0.0005 | -1.431929113 |
| Cyp2c55 | Mm.142581 | 72082 | 0 | -0.962281466 |
| 2700094K13Rik | Mm.259293 | 72657 | 0 | -1.002929998 |
| Dis3 | Mm.163339 | 72662 | 0 | -0.605882055 |
| Fezf1 | Mm.55115 | 73191 | 0.0001667 | -0.853113559 |
| 1700060L04Rik | | 73378 | 0.0003333 | -0.96950133 |
| 1700054N08Rik | Mm.157746 | 73420 | 0.0003333 | -0.64367993 |
| Acsl3 | Mm.276016 | 74205 | 0 | -0.907094599 |
| Morc4 | Mm.474566 | 75746 | 0.0001667 | -0.691308881 |
| Ncapg2 | Mm.21516 | 76044 | 0 | -0.802321707 |
| Cpb1 | Mm.34692 | 76703 | 0.0036667 | -0.852944194 |
| C030003D03Rik | Mm.266260 | 77220 | 0.0005 | -0.807160048 |
| 4930583I09Rik | Mm.109380 | 78057 | 0 | -1.244160177 |
| 4432416J03Rik | Mm.31626 | 78252 | 0.0005 | -0.867353981 |
| Apobec3 | Mm.284059 | 80287 | 0 | -0.628189902 |
| Trim2 | Mm.44876 | 80890 | 0.0001667 | -0.606036845 |
| Cenpq | Mm.9870 | 83815 | 0.0015 | -0.673545448 |
| Hmgb2 | Mm.279998 | 97165 | 0 | -0.791620614 |
| Psph | Mm.271784 | 100678 | 0 | -1.070754926 |
| Spon2 | Mm.34694 | 100689 | 0.0013333 | -0.828318476 |
| Trim68 | Mm.39043 | 101700 | 0.0001667 | -0.592433484 |
| Ces3 | Mm.292803 | 104158 | 0.0023333 | -0.89223621 |
| Akr1c18 | Mm.41337 | 105349 | 0.0048333 | -0.729479278 |
| Rfc4 | Mm.386835 | 106344 | 0.0001667 | -0.655545359 |
| Cyp2c50 | Mm.476076 | 107141 | 0.0013333 | -0.806315252 |
| Psat1 | Mm.289936 | 107272 | 0.0001667 | -0.865603326 |
| Cdc20 | Mm.289747 | 107995 | 0.0041667 | -0.658735542 |
| Slc22a7 | Mm.387538 | 108114 | 0 | -0.804299223 |
| Nusap1 | Mm.290015 | 108907 | 0.0003333 | -0.942815646 |
| Cdca2 | Mm.33831 | 108912 | 0.001 | -0.65715279 |
| Ela1 | Mm.2131 | 109901 | 0 | -0.736076829 |
| Amy2-1 | Mm.439729 | 109960 | 0.0001667 | -0.693523402 |
| Dek | Mm.131150 | 110052 | 0.0001667 | -0.61103604 |
| Cse1l | Mm.22417 | 110750 | 0.0001667 | -0.592169188 |
| BC089597 | Mm.214923 | 216454 | 0 | -1.019172876 |
| BC030867 | Mm.247335 | 217216 | 0.0016667 | -0.721495648 |
| Zfyve16 | Mm.259585 | 218441 | 0 | -1.453906415 |
| C330027C09Rik | Mm.24491 | 224171 | 0.0025 | -0.722717974 |
| Sfrs7 | Mm.292016 | 225027 | 0 | -0.643329636 |
| Cyp2c70 | Mm.29119 | 226105 | 0.0001667 | -0.879091016 |
| Zranb3 | Mm.440937 | 226409 | 0.0003333 | -0.592617029 |
| Pms1 | Mm.60499 | 227099 | 0.0011667 | -0.668888006 |
| 5430407P10Rik | Mm.133542 | 227545 | 0 | -1.041159139 |
| Cenpe | Mm.161470 | 229841 | 0 | -0.768428161 |
| BC023882 | Mm.319286 | 231123 | 0 | -0.689136847 |
| Cc2d2a | Mm.44434 | 231214 | 0.009 | -0.692353068 |
| Fkbp14 | Mm.274693 | 231997 | 0 | -0.658030879 |
| AI987944 | Mm.379390 | 233168 | 0 | -0.658595319 |
| BC015286 | Mm.443387 | 234669 | 0.0001667 | -0.94091953 |
| Ces2 | Mm.28191 | 234671 | 0.0003333 | -0.903115165 |
| Ces5 | Mm.441134 | 234673 | 0.0005 | -0.920976088 |
| Sc5d | Mm.32700 | 235293 | 0.001 | -0.749472746 |
| Topbp1 | Mm.259893 | 235559 | 0.0015 | -0.619846651 |
| Gas2l3 | Mm.11982 | 237436 | 0 | -1.16412344 |
| 4932411G14Rik | Mm.444776 | 238663 | 0 | -1.466639669 |
| Zfp367 | Mm.300065 | 238673 | 0.0003333 | -0.708753762 |
| Mtnr1b | Mm.222631 | 244701 | 0 | -0.83978379 |
| Olfr460 | Mm.377469 | 258381 | 0 | -0.613706995 |
| Olfr1510 | Mm.377492 | 258423 | 0.0026667 | -0.844928199 |
| Olfr771 | Mm.223077 | 258540 | 0.0006667 | -0.871486429 |
| Olfr491 | Mm.223164 | 258731 | 0 | -0.872676167 |
| Olfr812 | Mm.337275 | 258791 | 0 | -0.960527497 |
| Ccnb1 | Mm.260114 | 268697 | 0 | -1.213844596 |
| Prr11 | Mm.132381 | 270906 | 0.0025 | -0.596557084 |
| Afm | Mm.348786 | 280662 | 0 | -0.769933923 |
| 9930021D14Rik | Mm.258355 | 319259 | 0 | -0.596311116 |
| Tmem195 | Mm.476054 | 319660 | 0 | -0.703227908 |
| 5830418K08Rik | Mm.218317 | 319675 | 0.0006667 | -0.591544996 |
| B430105G09Rik | | 320907 | 0 | -0.783943754 |
| Pcdhac1 | Mm.308500 | 353236 | 0.0031667 | -0.660751738 |
| Raet1e | Mm.440891 | 379043 | 0 | -0.676616869 |
| 2700089E24Rik | Mm.291762 | 381820 | 0 | -0.606319097 |
| Tas2r130 | Mm.377916 | 387355 | 0 | -0.95657076 |
| LOC629242 | | 629242 | 0.0001667 | -0.961172381 |
| LOC100040402 | | 100040402 | 0.0008333 | -0.747221928 |
| ENSMUSG00000074179 | Mm.475927 | 100042314 | 0.0048333 | -0.817339681 |
| LOC100045864 | | 100045864 | 0.0008333 | -0.847903815 |
| 4921525D07Rik | |  | 0 | -1.340041553 |
| 5330425B07Rik | |  | 0 | -0.798968799 |
| IGKV2-105 | |  | 0.0013333 | -0.815122978 |
