# Supplementary material for: Loss of c-Met Disrupts Gene Expression Program Required for G2/M Progression during Liver Regeneration in Mice
Source: PLoS One. 2010 Sep 16;5(9):e12739. doi: 10.1371/journal.pone.0012739 (PMC2940888; doi:10.1371/journal.pone.0012739)
Supplement: Table S2 — List of antibodies. (0.05 MB DOC) [file pone.0012739.s007.doc]

**Table S2.**  List of antibodies

| **Antibody** | **Source** | **Cat. #** |
| --- | --- | --- |
| β−Actin | Chemicon | MAB1501 |
| AKT | Cell Signaling | 9272 |
| α-Tubulin | Sigma | T5168 |
| Aurora A | Novus Biologicals | ab12324 |
| Aurora B | Novus Biologicals | NB-100-294 |
| cdc2 | Cell Signaling | 9112 |
| cdcp34 | Santa Cruz | sc-239 |
| CENPA | Abcam | ab13939 |
| c-met | Santa Cruz | sc-162 |
| EGFR | Upstate | 06-129 |
| FOXM1 | Santa Cruz | sc-26688 |
| Histone H3 | Cell Signaling | 9715 |
| INCENP | Sigma | 15283 |
| MAD1 | Gift from K-T Jeang |  |
| MAD2 | BD Transduction | 6110678 |
| p38 | Cell Signaling | 9212 |
| p44/42 | Upstate | 05-157 |
| P-AKT  (Ser 473) | Cell Signaling | 9271S |
| P-cdc2  (Tyr15) | Cell Signaling | 9111 |
| P-c-Jun  (Ser 63) | Cell Signaling | 9261S |
| PCNA | Oncogene | NA03 |
| P-EGFR  (Tyr 1068) | Cell Signaling | 2234S |
| P-Histone H3  (Ser 10) | Cell Signaling | 9701S |
| PLK-1 | Zymed | 37-7100 |
| P-p38  (Thr180/Tyr182) | Cell Signaling | 4631S |
| P-p-44/42 | Cell Signaling | 9102 |
| P-SAPK/JNK (Thr183/Tyr 185) | Cell Signaling | 9251S |
| p-Stat3  (pY705) | BD Transduction | 612356 |
| P-Stathmin  ( Ser 16) | BioLegend | 620201 |
| Stat3 | BD Transduction | 610189 |
| Stathmin | Cell Signaling | 3352 |
| TGF-α | Neomarkers | MS-670-PO |
